# Efficacy of Radiotherapy for Oligometastatic Lung Cancer and Irradiation Methods Based on Metastatic Site

**DOI:** 10.3390/cancers17152569

**Published:** 2025-08-04

**Authors:** Katsuyuki Shirai, Masashi Endo, Shuri Aoki, Noriko Kishi, Yukiko Fukuda, Tetsuo Nonaka, Hitoshi Ishikawa

**Affiliations:** 1 Department of Radiology, Jichi Medical University Hospital, Shimotsuke 329-0498, Japan; r1707em@jichi.ac.jp (M.E.); y.fukuda@jichi.ac.jp (Y.F.); 2 Department of Radiation Oncology, The Cancer Institute Hospital, Japanese Foundation for Cancer Research, Tokyo 135-8550, Japan; shuri.aoki@jfcr.or.jp; 3 Department of Radiation Oncology and Image-Applied Therapy, Graduate School of Medicine, Kyoto University, Kyoto 606-8507, Japan; kishin@kuhp.kyoto-u.ac.jp; 4 Department of Radiology, Jichi Medical University Saitama Medical Center, Saitama 330-8503, Japan; 5 Department of Radiation Oncology, Japanese Red Cross Medical Center, Tokyo 150-8935, Japan; nonaka_tetsuo@med.jrc.or.jp; 6 QST Hospital, National Institutes for Quantum Science and Technology, Chiba 263-8555, Japan; ishikawa.hitoshi@qst.go.jp

**Keywords:** oligometastatic disease, lung cancer, radiotherapy, SBRT, surgery

## Abstract

Systemic chemotherapy is a standard treatment for patients with stage IV cancer with distant metastases, and there is little evidence of the effectiveness of local treatments for distant metastatic lesions. However, in recent years, randomized phase II trials targeting oligometastases in lung cancer and solid tumors have reported that local therapy combined with systemic chemotherapy improves clinical outcomes. We reviewed previous clinical trials and demonstrated the efficacy of radiotherapy for oligometastatic disease. Stereotactic body radiotherapy (SBRT) is a promising treatment that achieves high local control rates for oligometastatic disease. Although SBRT generally does not cause severe adverse events, the safety of SBRT combined with systemic chemotherapy needs to be carefully considered. We discussed the efficacy and safety of SBRT and summarized the details of SBRT methods and techniques for each metastatic site. Further research and clinical trials are warranted to improve the efficacy of SBRT combined with systemic chemotherapy for oligometastatic non-small cell lung cancer (NSCLC).

## Simple Summary

Prospective comparative studies have reported that local treatment, including radiotherapy and surgery, improves the survival of patients with oligometastatic disease. In particular, stereotactic body radiotherapy (SBRT) for oligometastatic diseases is a promising treatment that can achieve high local control rates without causing severe adverse events. However, radiation methods and techniques for each metastatic site, such as the lung, liver, bone, adrenal gland, lymph nodes, and brain, have not been fully established. In this article, we reviewed the clinical trials and showed the efficacy of SBRT for oligometastatic diseases. Furthermore, we summarized the recent SBRT methods and techniques for several metastatic sites. We hope that this manuscript will be helpful for the use of SBRT in the treatment of oligometastatic lung cancer.

## 1. Introduction

Systemic therapy is a standard treatment for patients with stage IV lung cancer with distant metastases. Whether local treatment can improve the outcomes of patients with oligometastatic disease—defined as cancer with a limited number of distant metastases—has been controversial. Furthermore, the disease concept and definition of oligometastases have not yet been fully established. The concept of oligometastases was proposed by Hellman & Weichselbaum in 1995, describing metastases to one or a small number of other organs in addition to the primary tumor [1]. They proposed that cancer comprises a biologic spectrum extending from a disease that remains localized to one that is systemic when first detectable but with many intermediate states. They suggested that oligometastases include two distinct clinical scenarios: (1) an early-stage of metastatic progression with a limited number of metastases, and (2) cases where multiple metastases were originally present but have been almost completely eradicated by systemic chemotherapy. To standardize inclusion criteria in future clinical trials, the European Organization for Research and Treatment of Cancer (EORTC) issued a multidisciplinary consensus statement on the definition of oligometastatic disease of lung cancer [2]. According to this statement, oligometastatic disease is defined as a condition in which radical treatment is technically feasible for all tumor sites with acceptable toxicity, and a maximum of five metastases in three organs is identified as the threshold.

In recent years, prospective comparative studies have reported that local treatment (such as surgery and radiotherapy) improves the survival of patients with oligometastatic disease. Stereotactic body radiotherapy (SBRT) for oligometastatic diseases is a promising treatment that can achieve high local control rates without causing severe adverse events. Based on these promising results, SBRT for oligometastases (≤five metastases) has been covered by insurance in Japan since 2020. In this article, we review clinical trials and demonstrate the efficacy of radiotherapy in oligometastatic diseases. Furthermore, we summarize the irradiation methods and techniques for each metastatic site, including the lung, liver, bone, adrenal gland, lymph nodes, and brain. A narrative review was performed to synthesize and interpret existing research in this manuscript.

## 2. Clinical Trials for Oligometastases of Lung Cancer

In recent years, randomized phase II studies have demonstrated the effectiveness of local treatments (such as surgery and radiation therapy) for oligometastases in lung cancer, which has completely changed the treatment strategy for this disease. In 2016, Gomez et al. reported a randomized phase II study that included 49 patients with stage IV non-small cell lung cancer (NSCLC) with oligometastases (≤three metastases) who had maintained stable disease for >3 months after systemic chemotherapy [3]. Inclusion criteria were the following: patients had a diagnosis of pathologically confirmed NSCLC, stage IV disease according to the 7th edition of the American Joint Committee on Cancer staging system, three or fewer metastases, not including the primary tumor, a performance status score of 2 or less, were 18 years or older, and had received standard first-line systemic therapy, defined as four or more cycles of platinum doublet chemotherapy, erlotinib or another approved first-line epidermal growth factor receptor (EGFR) tyrosine-kinase inhibitor for 3 months or longer if the patient was known to harbor an *EGFR* mutation, or crizotinib for 3 months or longer if the patient was known to have an *ALK* rearrangement. The exclusion criterion was having bevacizumab within 2 weeks of the initiation of radiotherapy. The study compared a group that received chemotherapy plus local treatment (such as surgery, SBRT, or conventional fractionated radiation therapy) for metastatic lesions with a group that continued chemotherapy alone. The median progression-free survival (PFS) was 11.9 months in the local therapy group and 3.9 months in the maintenance therapy group, demonstrating a significant improvement in PFS with local therapy (hazard ratio [HR]: 0.35, 90% confidence interval [CI]: 0.18–0.66, *p* = 0.0054). In a long-term follow-up published in 2019, the median survival time was 41.2 months in the local treatment group and 17.0 months in the maintenance therapy group, demonstrating improved overall survival (OS) with local treatment (*p* = 0.017) [4]. In 2018, Iyengar et al. reported a randomized phase II study including 29 patients with stage IV NSCLC and up to six oligometastases after four to six courses of platinum-based combination chemotherapy [5]. In this study, patients were eligible if they were 18 years or older, had a Karnofsky Performance Status score of 70 or better, and had biopsy-proven metastatic NSCLC. Patients must have received four to six cycles of first-line platinum-based chemotherapy, achieving stable disease or a partial response. Those receiving first-line targeted therapy for *EGFR*- positive and/or *ALK*-positive NSCLC were excluded. Patients were allowed to have up to 6 sites of extracranial disease (including primary) with no more than 3 sites in the liver or lung identified by diagnostic CT, PET-CT, or magnetic resonance imaging prior to enrollment. Individuals were ineligible if previously irradiated primary disease progressed within 3 months of that treatment. Patients with untreated and/or uncontrolled brain metastases or disease involving the gastrointestinal tract and skin were ineligible.

The study compared patients who received chemotherapy plus SBRT for metastatic lesions with those who continued chemotherapy alone. The median PFS was 9.7 months in the SBRT group and 3.5 months in the maintenance chemotherapy group, demonstrating a significant improvement in disease-free survival with SBRT (*p* = 0.01). In 2019, Palma et al. conducted a randomized phase II study (SABR-COMET) involving 99 patients with various stage IV solid tumors and up to five metastases in three organs, who had maintained stable disease for ≥3 months with standard-of-care therapy [6]. Patients were required to be aged 18 years or older, with good performance status, and a life expectancy of at least 6 months, as judged by the enrolling physician. Their primary tumor must have been treated definitively at least 3 months before enrolment, with no progression at that site since the definitive treatment as established by imaging. Patients were required to be discussed at a multi-disciplinary tumor board or quality assurance rounds before randomization, with consensus opinion that entry into the study was appropriate. Biopsy of a metastasis was not required but was preferred. All metastatic lesions had to be amenable to SBRT, and a maximum of three metastases in any one organ was allowed with no more than five metastases in total. The main exclusion criteria included serious medical comorbidities precluding radiotherapy, bone metastasis in a femoral bone, the presence of one to three brain metastases with no disease elsewhere, previous radiotherapy to a site requiring treatment, malignant pleural effusion, tumor within 3 mm of spinal cord on MRI, dominant brain metastasis requiring surgical decompression, pregnancy, or lactation. This study included 18 patients with lung cancer (18%) and compared the addition of SBRT to standard-of-care therapy with standard-of-care therapy alone. The median PFS was 12 months in the SBRT group and 6.0 months in the standard-of-care group, showing a significantly better outcome in the SBRT group (HR: 0.47, 95% CI: 0.30–0.76, *p* = 0.0012). The median survival time was 41 months in the SBRT group and 28 months in the standard-of-care group, with a trend toward improved OS in the SBRT group (HR: 0.57, 95% CI: 0.30–1.10, *p* = 0.090). The SABR-COMET trial also reported long-term data with extended follow-up, and median follow-up was 51 months [7]. The 5-year OS rates were 42.3% in the SBRT group and 17.7% in the standard care group, respectively (*p* = 0.006). There were no new grade 2–5 adverse events during extended follow-up. The authors concluded that the impact of SBRT on OS was larger in magnitude than in the initial analysis and durable over time, and there were no new safety signals in SBRT group. There are few papers reporting the long-term clinical outcomes of SBRT for oligometastases; therefore, further studies are warranted to show long-term efficacy and safety. In these previous studies, chemotherapy was the primary systemic therapy combined with local treatment; furthermore, the concurrent use of tyrosine kinase inhibitors (TKI) and SBRT for patients with EGFR-mutated NSCLC was reported in 2023 [8]. The randomized phase III study included 133 patients with EGFR-mutated NSCLC and synchronous oligometastatic disease. It compared SBRT in combination with a TKI versus TKI monotherapy. The combination therapy significantly improved median PFS (20.2 months vs. 12.5 months, *p* < 0.001) and median survival time (25.5 months vs. 17.4 months, *p* < 0.001).

There are few papers on the impact of SBRT for oligometastatic disease on quality of life (QOL). In the SABR-COMETS trial, a secondary analysis was conducted to evaluate changes in QOL after SBRT [9]. QOL in the SBRT and the standard-of-care groups were assessed using the Functional Assessment of Cancer Therapy-General. The authors concluded that average QOL declines slowly over time regardless of treatment approach, and the use of SBRT was not associated with a QOL detriment compared with standard-of-care. These trials share the common feature of focusing on patients with synchronous oligometastatic disease and comparing groups receiving combined systemic and local therapy versus systemic therapy alone. All reported that the addition of local therapy—particularly SBRT—significantly improved the OS and PFS. In the future, combinations of various systemic therapies with SBRT are expected to offer effective treatment strategies for patients with oligometastatic diseases. Furthermore, clinical studies are warranted to demonstrate the efficacy of SBRT for other type of oligometastases, such as metachronous, repeated, or induced oligometastases.

## 3. Pulmonary Oligometastasis of Lung Cancer

Pulmonary metastasis is one of the most common forms of cancer metastasis, originating from various primary malignancies such as lung, head and neck, breast, kidney, colorectal, and gynecological cancers, as well as sarcomas. The predominant route of spread for pulmonary metastasis is hematogenous. The incidence of pulmonary metastasis varies by primary tumor histology, ranging from 20% to 50%, and the frequency of metastases confined to the lungs has been reported to range from 0% to 25% [10]. The SABR-COMET trial reported that 53% of patients in the standard-of-care group and 43% in the standard-of-care plus SBRT group had pulmonary oligometastases [7]. In both groups, primary lung cancer accounted for 18% of cases, followed by colorectal cancer. These findings suggest that pulmonary oligometastases, particularly those originating from lung cancer, play a considerable role in shaping clinical strategies for oligometastatic disease.

Previous studies on pulmonary metastases have investigated the efficacy and toxicity of local therapies, such as SBRT, surgical metastasectomy, and percutaneous thermal ablation, although no randomized phase III trials have directly compared these treatment modalities. Current comparative evidence is derived from retrospective studies, some of which have used propensity score-based methods to mitigate treatment selection bias. Moreover, these reports often included heterogeneous populations, such as patients with pulmonary oligometastases originating from various primary tumors other than NSCLC. Representative reports comparing different modalities for treating pulmonary metastases are presented in Table 1. SBRT has achieved comparable local control rates with surgery and been considered a promising non-invasive alternative, particularly for elderly patients or those with comorbidities who are unsuitable surgical candidates. A retrospective study comparing sublobar resection, SBRT, and thermal ablation reported 2-year cumulative incidences of local recurrence of 9.6% for sublobar resection, 11.7% for thermal ablation, and only 4.1% for SBRT [11]. However, SBRT may offer inferior local control for pulmonary metastases originating from colorectal cancer, renal cell carcinoma, or sarcoma compared to surgery or thermal ablation [12,13]. Collectively, these findings suggest that the optimal local therapy for pulmonary metastases depends on the primary tumor type, patient characteristics, and anticipated course of disease control.

In the previously reported randomized controlled phase II trials, which predominantly using SBRT techniques, site-specific outcomes based on metastatic location were not clearly described. However, all included a substantial proportion of patients, approximately 80% to 93%, with pulmonary oligometastases or primary lung tumors [4,5]. Tsai et al. evaluated standard-of-care systemic therapy with or without SBRT in patients with oligoprogressive breast cancer and NSCLC [18]. In this trial, an addition of SBRT to the standard-of-care contributed to more than a four-fold increase in PFS. Pulmonary tumors accounted for 26% of all treated lesions (including both breast cancer and NSCLC cases). The median PFS in the SBRT group was 10.0 months. Although the proportion of pulmonary oligometastases was not specified, 16% of patients in the SBRT group experienced ≥ grade 2 toxicities, including radiation pneumonitis and brachial plexopathy.

These phase II studies used a wide range of fractionation regimens for pulmonary oligometastases, reflecting the current lack of a standardized approach. The optimal SBRT fractionation schedule varies depending on several factors, including the location, number, and size of pulmonary oligometastases; presence of nodal metastases; and concurrent use of systemic therapy. In the study by Gomez et al., fractionation regimens ranged from conventional fractionation (66 Gy in 33 fractions with concurrent chemotherapy) to highly conformal SBRT doses [3]. Iyengar et al. utilized a range of SBRT regimens, including 21–27 Gy in a single fraction, 26.5–33 Gy in three fractions, 30–37.5 Gy in five fractions, and 45 Gy in fifteen fractions [5]. Tsai et al. primarily used regimens of 27–30 Gy in three fractions or 30–50 Gy in five fractions [18]. All dose and fractionation regimens were selected using a risk-adapted approach to achieve an appropriate balance between treatment efficacy and safety. Regarding SBRT fractionation for 1–3 peripherally located pulmonary oligometastases, the SAFRON II trial, a randomized phase II study, compared single- and multifraction regimens in a cohort that included 11% of pulmonary oligometastases from NSCLC and found no considerable differences in safety, efficacy, or survival between the two arms [19]. In addition to comparable clinical outcomes, the single-fraction has been shown to have lower initial costs compared to multifraction regimens and is highly likely to be cost-effective, as demonstrated in a prespecified analysis of the SAFRON II trial, with an incremental cost-effectiveness ratio of Australian dollars (AUS) 15,821/quality-adjusted life-years (QALY) within 4 years and a 97% probability of cost-effectiveness at a threshold of A$50,000/QALYs [20]. For bulky tumors or lesions adjacent to the chest wall, multifraction regimens are more appropriate because of concerns about local control and potential toxicity. Several previous studies have demonstrated an association between biologically effective dose (BED) and local control, suggesting that, when feasible, a minimum BED of 100 Gy should be targeted to optimize tumor control [21]. Although dose escalation to the planning target volume (PTV) may enhance local control, it must be balanced with the risk of toxicity, particularly in centrally located pulmonary oligometastases. Increased radiation dose to organs at risk, such as the trachea, proximal bronchial tree, esophagus, heart, major vessels, spinal cord, brachial plexus, phrenic nerve, and recurrent laryngeal nerve, has been associated with a higher incidence of severe adverse events. Therefore, the use of SBRT as definitive local therapy in such cases should be carefully individualized. For pulmonary metastases located in ultracentral regions, where the PTV abuts the mediastinum and critical organs at risk, de-escalation of the SBRT dose to a BED of ≥75 Gy may be a safer approach. Furthermore, when SBRT is administered in combination with systemic therapy, dose de-escalation below a BED of 100 Gy should be considered to mitigate potential toxicities. As no high-level evidence currently guides clinical management regarding the combination of modern targeted therapies or immunotherapies with SBRT, careful consideration, such as avoiding concurrent administration or implementing a treatment pause based on the half-life of the systemic agent is warranted to minimize the risk of adverse events [22]. Therefore, SBRT is a feasible treatment option for patients with multiple pulmonary oligometastases. Both synchronous SBRT for multiple lesions and repeat SBRT have demonstrated safety and efficacy profiles comparable to those observed in patients treated for a single pulmonary oligometastasis. The incidence of early mortality within 3 or 6 months did not increase in patients who underwent multiple SBRT courses, and no grade 4 or 5 toxicities were reported in this population [23]. In parallel with SBRT, particle beam therapy is also emerging as a promising modality. It is characterized by high biological effectiveness and excellent dose conformity. This enables for reduced radiation exposure to mediastinal organs at risk adjacent to central lung tumors, or to the lung in high-risk cases such as those with interstitial pneumonia. While clinical experience is accumulating, evidence for its use in metastatic lung tumors remains limited, and further development is anticipated.

In summary, SBRT has achieved high local control rates and been considered a promising non-invasive local therapy for pulmonary oligometastases. However, current evidence is primarily derived from phase II trials and retrospective studies involving heterogeneous cohorts, and the existing data remains insufficient. Particle beam therapy may also be beneficial in selected high-risk cases due to its superior dose distribution. Multidisciplinary discussion, including comparative evaluation with other local modalities, should be undertaken to develop appropriate strategies tailored to individual patient characteristics and tumor-specific factors.

## 4. Liver Oligometastases of Lung Cancer

This section discusses the use of SBRT in the management of liver metastases from lung cancer, including its clinical role, treatment methods, efficacy, and adverse events. The liver is a common site of metastasis in most solid malignancies, including lung cancer. Liver metastases have been reported in 40% of lung cancer autopsies. Clinically, liver metastases are among the most common sites of initial metastases, accounting for 10–20% of cases of NSCLC [24,25,26] and 30–50% of cases of small cell lung cancer (SCLC) [27,28]. Conversely, in a prospective study of synchronous oligometastases in NSCLC, Gomez et al. reported liver metastases in only 2 of 49 patients (4%) [3]. In lung cancer, the paucity of reports on local therapy for liver oligometastases compared with the overall incidence is likely due to the high initial prevalence of multiple or multi-organ metastases and the longstanding emphasis on systemic treatment with a variety of drug regimens [26]. Most previous reports on radiotherapy for liver oligometastases have focused on colorectal cancer; however, there are shared findings regarding the safety and efficacy of radiotherapy for primary tumors.

Surgical resection remains the first-line local therapy for achieving long-term survival in patients with a limited number of liver metastases. However, only 10–30% of liver metastases are eligible for surgical resection [29], prompting the exploration of alternative therapies such as radiofrequency ablation, transcatheter arterial chemoembolization, and radiotherapy [30]. The role of radiotherapy in liver tumors has historically been limited due to the high radiosensitivity of the organ [31]; however, with the development of SBRT, it has become possible to deliver high doses to tumors while sparing normal liver tissue. Prospective and retrospective studies on SBRT for liver oligometastases have shown promising results in terms of local control, OS, and toxicity. Reports specifically on liver metastases from lung cancer are summarized in Table 2. SBRT has been defined as an alternative modality for unresectable or post-resection isolated liver metastases in most clinical trials because of its greater flexibility than surgery in terms of tumor location and constraints related to vascular invasion or peritoneal involvement [32]. The most common indications for SBRT for liver metastasis include a maximum tumor diameter of <6 cm, controlled or absent extrahepatic disease, ≤five lesions, good performance status (Eastern Cooperative Oncology Group [ECOG] 0–1 or Karnofsky Performance Status > 70), and adequate liver volume and function [33,34]. Age was not used as a selection criterion in most studies. Several reports have suggested that SBRT achieves better tumor control in lesions ≥ 2 cm [35] or ≥3 cm [36] in diameter than radiofrequency ablation. However, tumor control with SBRT tends to be reduced for large tumors (≥5 cm) [37]. Particle beam therapy, which offers a more conformal dose distribution [38,39], is considered for bulky tumors and tumors located in the caudate lobe and hepatic hilum; however, in Japan it is not covered by insurance for metastatic tumors, and the cost issues remain.

**Figure 1:**
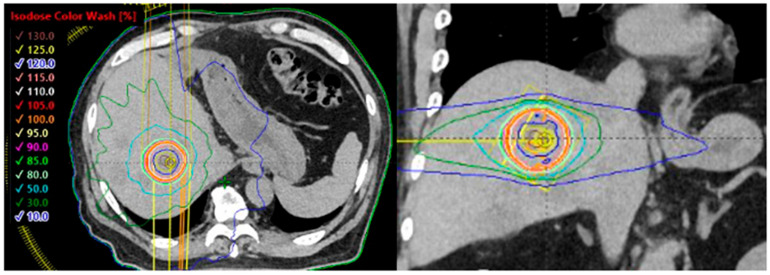
Dose distribution of stereotactic body radiotherapy (SBRT) for liver metastases using volumetric modulated arc therapy (VMAT). The central dose is approximately 130% for a prescription of 40 Gy in five fractions to 95% of the planning target volume (PTV). The red/orange/yellow lines representing the 105%, 100%, and 95% dose lines are concentrated around the PTV contour (red line).

Liver tumors are often difficult to accurately identify using plain computed tomography (CT) alone; therefore, multimodal imaging using contrast-enhanced CT and magnetic resonance imaging (MRI) should be employed for treatment planning. The target should be appropriately defined by fusing planning CT images with those from diagnostic imaging. Liver tumors tend to exhibit greater respiratory motion than lung tumors; therefore, treatment planning should aim to minimize the target size using techniques such as four-dimensional CT and delivery methods like respiratory gating or breath-hold techniques. In addition, dietary restrictions may be required approximately 3 h before treatment owing to gastrointestinal influences. Baseline shifts during irradiation can exceed 1 cm, necessitating appropriate image guidance and adjustments during treatment. The placement of one or more gold fiducial markers on the liver adjacent to the tumor is useful for obtaining accurate positional information during irradiation and for reducing the treatment target, which is essential for motion-tracking irradiation. An example of dose distribution of liver SBRT is shown in Figure 1. Currently, there are no standardized guidelines for the prescribed doses or fractionation schedules in liver SBRT. Appropriate regimens vary depending on tumor size, location, and proximity to organs at risk. Reported SBRT regimens generally involve doses ranging from 25 to 75 Gy in 3–6 fractions. The most commonly used schedule is three fractions [32,33,42], whereas in Japan, 42–48 Gy in four fractions is the most prevalent regimen [43]. Previous prospective and retrospective studies of patients with oligometastatic liver metastases have demonstrated that SBRT can achieve 2-year local control rates of 60–100% (Table 2) [33,34,40,41,42]. The relationship between prescribed dose and tumor control is well established, and several publications recommend delivering a BED (α/β = 10 Gy) ≥ 100 Gy as a threshold for achieving adequate local control [37,40,42]. In a study by Kok et al., the 2-year OS for patients receiving a BED (α/β = 10 Gy) ≤ 100 Gy and >100 Gy were 48% and 85%, respectively [44]. Other studies have used an equivalent dose in 2 Gy fractions >150 Gy as a benchmark [45]. Several prospective studies have also explored single-fraction SBRT [41,46]. Folkert et al. reported excellent local control with single-fraction regimens 35–40 Gy, achieving a 4-year local control rate of 96.6% [44].

One prognostic factor for liver metastases from lung cancer is the site of the primary tumor [30]. Previous studies have reported both favorable [45] and unfavorable [47] local control outcomes for lung cancer compared with colorectal cancer and other cancers. Variability in treatment response has also been linked to genetic mutations [48]. In the future, treatment may be increasingly personalized based on differences in tumor radiosensitivity and concurrent systemic therapy. Additional tumor-related factors associated with poor prognosis include tumor size (>3 cm) [34,37,43], number and total volume of liver metastases [42], and history of prior chemotherapy [30]. Numerous studies have shown that achieving local control with SBRT contributes to improved survival in patients with liver oligometastases [42,49]. However, some reports suggested that lung cancer is often associated with a poorer prognosis owing to the development of distant metastases [33,42,50]. Further studies are warranted to investigate the potential synergistic effects of combined SBRT with immunotherapy or targeted therapy, although appropriate combination strategies and dosing regimens remain under discussion. The influence of oligometastatic timing on clinical outcomes has not been thoroughly examined. Although some surgical series suggest that metachronous oligometastases are associated with better local control and OS than synchronous lesions [51], other reports on SBRT indicate no difference [52].

Severe toxicity associated with SBRT for oligometastatic liver metastases is rare. Most previous reports have shown grade ≥ 3 toxicity rates of 0–10% [32,33,34,42], indicating a favorable toxicity profile. Radiation-induced liver disease (RILD) is a clinical syndrome that typically presents 2 weeks to 3 months after radiation therapy, characterized by anicteric hepatomegaly, ascites, and elevated liver enzymes. In most cases, RILD improves with observation alone, and the incidence of severe RILD following SBRT has been reported to be <1% [32,33]. To prevent RILD, careful pre-treatment assessment of liver function is essential, and treatment plans with lower doses to normal liver tissue are recommended (e.g., cumulative total tumor volume of 700 cm^3^ or more of healthy liver tissue with a cumulative total dose of less than 15 Gy in three fractions or less than 9.1 Gy in a single fraction). Other common grade 2 toxicities include gastrointestinal, soft tissue, and bone complications, particularly in lesions close to the duodenum, bowel, skin, and ribs [30]. Dose constraints apply to at-risk organs such as the stomach, duodenum, spinal cord, and kidneys. Several clinical trials have published dose regimens and constraints for these organs, which may serve as useful references [29,33,34,41]. In summary, based on current clinical evidence, SBRT for liver metastases has demonstrated both high safety with favorable local control, supporting the appropriateness of these dose constraints.

## 5. Adrenal Oligometastases of Lung Cancer

This section discusses the role of SBRT in managing adrenal oligometastases in lung cancer, focusing on treatment efficacy, delivery techniques, and clinical considerations. The adrenal glands are a common site of distant metastasis in patients with lung cancer, with involvement reported in approximately 42% of autopsy cases [53]. According to the International Association for the Study of Lung Cancer database, the frequency of solitary adrenal metastases in NSCLC is estimated to be approximately 20% [54]. Adrenal metastases are rarely symptomatic, occurring in about 4% [55], and are typically detected incidentally during staging or follow-up imaging, such as contrast-enhanced CT or 18-fluoro-deoxyglucose positron emission tomography (FDG-PET)/CT. Traditionally, systemic therapy has been the standard treatment approach for adrenal metastases in lung cancer, with palliative radiotherapy occasionally considered for symptomatic cases [56]. However, local therapies such as surgery and SBRT are being explored for patients with adrenal oligometastases [57,58]. Surgical resection offers high rates of local control but is associated with considerable risk. In a retrospective multicenter study, Metman et al. analyzed outcomes in 95 patients who underwent adrenalectomy for metastatic disease, including 25 with lung cancer, and reported a postoperative complication rate of 37.9% [59]. Regarding SBRT outcomes, a meta-analysis of retrospective studies on adrenal oligometastases (with lung cancer comprising 641 of 1006 cases [65.7%], and a median BED [α/β = 10 Gy]: 67 Gy) reported 1- and 2-year local control rates of 82% (95% CI, 74–88%) and 63% (95% CI, 50–74%), respectively. The 1- and 2-year OS were 66% (95% CI, 57–74%) and 42% (95% CI, 31–53%), respectively, with grade ≥3 adverse events reported in 1.8% of cases [58]. These findings suggest that SBRT may be a suitable treatment option for adrenal oligometastases from lung cancer. Although the number of retrospective studies has increased, prospective studies remain limited. Table 3 summarizes representative retrospective studies on SBRT for adrenal oligometastases from lung cancer [60,61,62,63,64]. Currently, there is no standardized dose-fractionation regimen for adrenal SBRT. Reported practices vary widely, including differences in prescription methods (such as isocenter, PTV, or isodose line based prescriptions). In the aforementioned meta-analysis, as well as in the reports by Zhao et al., Arcidiacono et al., and Rzazade et al. [62,63,64], higher tumor BEDs were associated with improved treatment outcomes. These findings suggest the importance of devising strategies to escalate target dose while maintaining safety.

**Figure 2:**
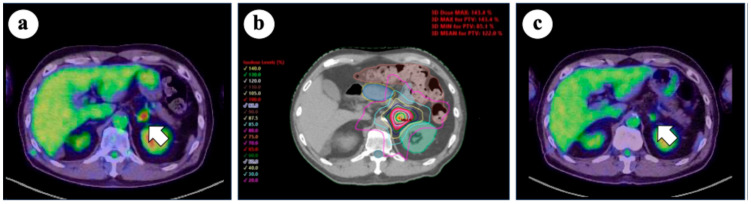
(**a**) 18-fluoro-deoxyglucose positron emission tomography/computed tomography (FDG-PET/CT) before stereotactic body radiotherapy (SBRT), showing increased FDG uptake in the left adrenal gland (arrow); (**b**) SBRT plan for the left adrenal metastasis. SBRT was prescribed with a dose of 40 Gy in five fractions to 95% of the planning target volume (PTV) and 70% isodose lines; (**c**) FDG-PET/CT scan 1 year after SBRT, demonstrating response in the treated lesion (arrow). No severe adverse events were observed.

Adrenal SBRT is particularly challenging because of the proximity of the adrenal glands to critical structures such as the upper gastrointestinal tract, liver, and kidneys. Therefore, careful treatment planning is essential to balance efficacy with safety. In Japan, a survey conducted by the Subcommittee of the High-Precision External Beam Radio-therapy Group of the Japanese Society for Radiation Oncology reported that a dose of 35–40 Gy in five fractions prescribed to 95% of the PTV has become a common practice [65]. When the target lesion is close to critical structures, a dose of 35 Gy in five fractions is often used to ensure safety. Based on this approach, prescribing the 60–80% isodose line to increase the tumor dose represents a practical method in clinical settings. Figure 2 shows the representative dose distribution of SBRT for adrenal oligometastases of lung cancer. Safe and effective delivery of adrenal SBRT requires attention to detail, including the use of four-dimensional CT, image-guided radiotherapy, breath-hold techniques, and tracking systems. In a meta-analysis by Liao et al. (involving 915 patients with lung cancer out of 1483 total cases [60.6%], median BED [α/β = 10 Gy]: 71.4 Gy), the use of tracking techniques was associated with improved OS. These findings highlight the importance of technological advancements in precision SBRT for adrenal metastases [66]. Although it remains uncertain whether these advanced SBRT techniques directly improve patient outcomes, further prospective studies are needed to clarify their impact. Nevertheless, the careful implementation of precision-guided techniques is essential for the safe and effective delivery of adrenal SBRT.

There are several clinical considerations that patients with adrenal oligometastatic lung cancer should be aware of when receiving SBRT. In many cases, these patients are either undergoing or being considered for various systemic therapies. As the safety of SBRT in combination with certain agents, particularly monoclonal antibodies, remains unclear, careful coordination among clinicians is essential when planning SBRT. The European Society for Radiotherapy and Oncology (ESTRO) and EORTC OligoCare project consortium have issued expert consensus recommendations regarding the timing of SBRT relative to various monoclonal antibodies, which should be consulted during clinical decision-making [22]. Specifically, for immune checkpoint inhibitors (ICIs), SBRT should be paused during treatment; however, no additional interruptions before or after SBRT are considered necessary. For anti-vascular endothelial growth factor agents, to minimize the risk of severe gastrointestinal toxicity, SBRT should be administered ≥1 week after drug administration, with a treatment break during SBRT and for ≥one additional treatment cycle afterward. Although concerns have been raised regarding SBRT-related adrenal insufficiency, few comprehensive reports exist on this issue. Studies analyzing cases of adrenal metastases before the widespread adoption of adrenal SBRT have found that 19–33% of patients with bilateral adrenal metastases exhibited signs of adrenal insufficiency. In addition, some patients with unilateral adrenal metastases detected on CT imaging also exhibited symptoms of adrenal insufficiency [67,68]. Moreover, Hamidi et al. reported that patients undergoing bilateral or unilateral adrenal SBRT with a history of contralateral adrenalectomy may be at increased risk of adrenal insufficiency [69]. In the modern management of lung cancer, adrenal insufficiency due to adrenal metastases or immune-related adverse events [70] should always be considered. In such cases, specialized evaluation by an endocrinologist may be warranted. However, data on the epidemiology and risk prediction of treatment-related adrenal insufficiency remain limited, highlighting the need for further research.

In summary, given its ability to achieve high local control rates with minimal toxicity, SBRT is a well-tolerated and effective treatment option for patients with lung cancer and adrenal oligometastases. Ongoing advancements in precision adrenal SBRT techniques, integration with systemic therapies, and research into endocrine manifestations will help further clarify the optimal management strategies for adrenal oligometastases in lung cancer.

## 6. Bone Oligometastases of Lung Cancer

Approximately 35–40% of patients with lung cancer develop bone metastases during their disease course [71]. Among patients with M1b NSCLC, approximately 35% have a single bone metastasis [54]. Although conventional radiotherapy for bone metastases has primarily been used for palliative purposes, the significance of local treatment for oligometastases has been increasingly recognized. SBRT allows the delivery of high radiation doses to tumors while minimizing exposure to organs at risk such as the spinal cord, making it a promising approach for achieving higher local control rates compared to conventional radiotherapy. SBRT also offers a non-invasive, short-course treatment option that can be easily integrated with systemic therapy, making it particularly suitable for patients with bone oligometastatic lung cancer. This section provides an overview of SBRT for bone oligometastatic lung cancer.

**Figure 3:**
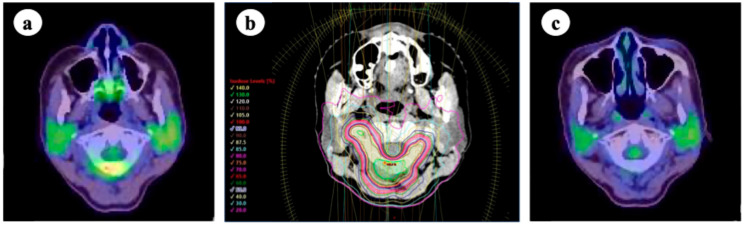
(**a**) 18-fluoro-deoxyglucose positron emission tomography/computed tomography (FDG-PET/CT) before stereotactic body radiotherapy (SBRT); (**b**) SBRT targeting the atlas (24 Gy in two fractions, prescribed to 95% of the planning target volume [PTV] and 70% isodose line); (**c**) FDG-PET/CT 1 year after SBRT. The lesion remained controlled without significant adverse events.

The efficacy of local therapy for oligometastatic lung cancer in improving prognosis and local control rates has been demonstrated in various clinical trials. In a randomized phase II trial conducted by Gomez et al. to evaluate the efficacy of local therapy for oligometastatic NSCLC, bone oligometastases were observed in 10 of 49 cases [3]. However, most patients receive conventional radiotherapy (30–45 Gy in 10–15 fractions). Currently, SBRT is often considered for the treatment of bone oligometastases. The SC.24 trial [72] was a randomized phase II/III comparative trial evaluating the efficacy of SBRT (24 Gy in two fractions) versus conventional radiotherapy (20 Gy in five fractions) in patients with painful spinal metastases, with lung cancer cases comprising 61 out of the 229 enrolled patients (26.6%). The primary endpoint, the complete pain response rate at 3 months, was significantly higher in the SBRT group (35% vs. 14%, *p* < 0.001), confirming its superiority. Subsequent long-term analyses demonstrated that the 2-year local control rate was significantly higher in the SBRT group than conventional radiotherapy (85% vs. 64%, *p* < 0.001), with no significant differences in adverse events between the groups [73]. The potential benefit of SBRT for bone oligometastases has also been highlighted in a multicenter phase III trial led by Zelefsky et al., which included 11 patients with lung cancer out of 117 (9.4%) [74]. A median follow-up period of 52 months indicated favorable long-term outcomes in this population. These results suggest favorable efficacy and safety of SBRT in the treatment of locally controlled bone oligometastases. Currently, there is no consensus regarding the optimal SBRT dose fractionation for local bone oligometastatic control. Zelefsky et al. investigated the local efficacy of two SBRT regimens for bone oligometastases: 24 Gy in a single fraction and 27 Gy in three fractions. Their findings showed a significantly higher local control rate in the single-fraction arm, with local recurrence rates at 2 and 3 years of 2.7% and 5.8%, respectively, compared with 9.1% and 22% in the three-fraction arm (*p* = 0.0048). Regarding spine SBRT, a meta-analysis by Soltys et al. showed that radiation doses of 18–24 Gy in a single fraction, 24 Gy in two fractions, 30 Gy in three fractions, 33 Gy in four fractions, and 35 Gy in five fractions all achieved 2-year local control rates > 80%, serving as a valuable reference for clinical practice [75]. A survey conducted by the Subcommittee of the High-Precision External Beam Radiotherapy Group of the Japanese Society for Radiation Oncology showed that 30–35 Gy in five fractions and 24 Gy in two fractions, prescribed to 95% of the PTV, are commonly used in clinical practice in Japan [65]. An example of dose distribution of SBRT for an atlas vertebra is shown in Figure 3. Regarding non-spine SBRT, a recent systematic review and meta-analysis conducted under the auspices of the International Stereotactic Radiosurgery Society reported favorable outcomes, with pooled local control rates of 95% at 1 year and 94% at 2 years [76]. In this report, the expert consensus recommended dose fractionation schedules of 18–24 Gy in a single fraction, 27–30 Gy in three fractions, and 30–35 Gy in five fractions. Although the panel supported dose escalation beyond 35 Gy up to 50 Gy in five fractions, the optimal maximum dose remains unclear owing to limited clinical data. Within these ranges, a higher dose was considered beneficial for improving local control; however, the prescribed dose should be carefully adjusted based on factors such as the proximity of organs at risk, prior radiation history, and anatomical considerations. Evidence specifically focusing on bone oligometastases from lung cancer remains limited, and further investigation is warranted to determine the optimal SBRT strategy in this subset. In this context, the results of the ongoing multicenter phase III trial (STEREO-OS trial), which evaluates the efficacy of SBRT for bone oligometastases in various solid tumors including NSCLC, may provide valuable clinical insights in the future [77].

Several technical challenges must be addressed when performing spinal SBRT. Radiation-induced myelopathy can lead to irreversible neurological deficits; its prevention is therefore of the utmost importance. Although various treatment approaches, including corticosteroids, bevacizumab, hyperbaric oxygen therapy, and anticoagulants, have been reported to offer potential benefit, none have been established as standard therapies [78,79]. Accordingly, precise spinal cord dose evaluation is essential and requires accurate spinal contouring on planning CT.

MRI is crucial because the spinal cord is difficult to visualize using CT alone. Based on their clinical experience with hybrid MRI and radiotherapy systems, Spieler et al. emphasize that performing MRI using the same immobilization conditions as those for CT planning ensures consistent spinal curvature across modalities, thereby facilitating accurate image fusion [80]. The spinal cord is delineated on MRI, and a 1.5–2.0 mm margin is added to define the planning organ-at-risk volume, which is used for dose constraints [81]. Target volume delineation in SBRT differs from that in conventional RT. Many ongoing clinical trials have adopted a consensus approach that divides the spine into anatomical compartments and defines the clinical target volume based on the involved compartment [82], which should be considered in clinical practice. In clinical practice, spinal SBRT planning often necessitates dose de-escalation to the target volume owing to the size of the tumor and its anatomical relationship with the spinal cord. Kowalchuk et al. reported that a compromised tumor dose was associated with decreased local control rates [83]. Accordingly, early detection of bone oligometastases is of critical importance, and timely initiation of SBRT planning should be considered to maximize the likelihood of durable local control.

This section summarizes the current status and key considerations of SBRT for local control of bone oligometastases. Although the optimal dose and fractionation schedule for different anatomical sites remain an area of ongoing investigation, SBRT enables controlled interventions for oligometastases, expanding therapeutic options for patients with local control.

## 7. Stereotactic Body Radiotherapy for Oligometastases of Lymph Node

In treating oligometastatic lesions using SBRT, tumor size and the characteristics of the primary disease (that is, cancer type and histology) are considered major factors influencing the local control of metastatic lesions. The performance status of the patient, number of metastatic lesions, and status of the oligometastatic disease, as proposed by the ESTRO and EORTC [84], are regarded as prognostic factors for survival rate [85]. Conversely, it has been reported that the lesion site has little effect on treatment outcomes [85,86]. Reflecting this background, no large-scale clinical trials or extensive case series focusing exclusively on lymph node oligometastases have been identified in the reviewed literature. The SABR-5 trial was a phase II study conducted in Canada that aimed primarily to evaluate the toxicity profile of SBRT in patients with 1–5 oligometastatic lesions [87]. A total of 381 patients (549 lesions) were enrolled, and treatment outcomes and prognostic factors were analyzed in addition to toxicity [85,87,88]. Baker et al. reported that 1- and 3-year local control rates for all lesions were 93% and 87%, respectively, and multivariate analysis revealed that colorectal histology and larger tumor size were significantly associated with inferior local tumor control [85]. Among these lesions, 78 lymph node metastases (14.2%) were included. Although these sites exhibited a trend toward better local control than other sites, this difference did not reach statistical significance. PFS was also analyzed in this study, and tumor size and a disease-free interval of <18 months were identified as significant factors on multivariate analysis. However, metastatic site was not a significant prognostic factor. Recently, Franceschini et al. reported the results of a single prospective observational phase II trial on ablative SBRT for medically inoperable thoracic lymph node metastases [89]. In this study, 32 patients (41 lesions) were included, and NSCLC was the most common primary tumor (13 patients, 40.6%). Notwithstanding the necessity of partial under coverage of PTV in 66% of cases, the local control rates at 1 and 2 years were 93.5% and 82.3%, respectively, with no acute or late toxicity ≥ grade 3.

In terms of the dose fraction of SBRT for lymph node metastases, a domestic survey conducted by Japanese Society for Radiation Oncology reported that 35–40 Gy in five fractions was the most commonly used regimen [65]. Similarly, in the SABR-5 trial, a dose of 40 Gy in five fractions was generally adopted for lymph node metastases [88]. Shahi et al. reported that SBRT using 35 Gy (range, 30–50 Gy) in five fractions had a 9.0% incidence of local recurrence at 2 years for mediastinal and hilar lymph node metastasis [90]. Regardless of the specific site of metastasis, the most critical aspect of SBRT for oligometastases is ensuring treatment safety through meticulous planning to avoid excessive radiation exposure to adjacent normal tissues. Particularly in the lymph node regions, tumors are often located close to critical normal organs. In such cases, prioritizing dose delivery to the tumor may pose a risk of exceeding the tolerance dose of the adjacent organs at risk. In a study by Franceschini et al., doses of 50 Gy in five fractions, 60 Gy in eight fractions, and 70 Gy in 10 fractions were employed depending on the location and number of lesions [89]. In cases where lesions were adjacent to critical normal organs, treatment was administered within the tolerance dose limits of these organs, and a reduction in the dose to the PTV was deemed acceptable. Cereno et al. evaluated the relationship between the planned and delivered dose to the PTV using the Coverage Compromise Index (CCI) in patients enrolled in the SABR-5 trial [91]. The mean CCI for all patients was 0.88. Lesions with a CCI < 0.90 (that is, delivered dose below the planned dose) comprised 196 lesions (36%), and comparison with those having a CCI ≥ 0.90 revealed no significant differences in local control or PFS.

Lymph node oligometastases selected for local therapy often include metachronous, recurrent, or induced lesions, with the latter emerging as oligometastases following systemic therapy for initially widespread disease. With the increasing availability of molecular targeting agents across various cancer types, their concurrent use with SBRT for treating lymph node oligometastases requires careful consideration. Recently, the OligoCare Project published a consensus statement on the concurrent use of SBRT and molecular targeting agents, including ICI [22]. It is important to recognize that this consensus should not be interpreted as a guideline or endorsement of concurrent administration but rather as a reference for clinical decision-making. The consensus required agreement by ≥75% of expert panel members; for drugs without consensus, individual expert opinions and their proportions should be reviewed. Among the agents evaluated, the authors strongly recommended avoiding the concurrent use of anti-vascular endothelial growth factor antibodies and multi-kinase inhibitors, such as sunitinib and sorafenib. In such cases, an interval of ≥1–2 weeks was maintained between drug administration and SBRT. In summary, SBRT for oligometastatic lymph node metastases is a promising treatment without severe adverse events. Prospective study is warranted to establish the safe and effective SBRT methods combined with systemic therapy for oligometastatic lymph node metastases.

## 8. Metastatic Brain Tumors of Lung Cancer

Brain metastases represent the most common site of distant spread in patients with lung cancer, with approximately 50% of patients with NSCLC developing brain metastases [92]. The primary goals of radiotherapy for brain metastases are to alleviate neurological symptoms, prevent central nervous system death, and improve survival and quality of life. Horton et al. reported in 1971 that whole-brain radiation therapy (WBRT) prolonged survival compared with steroid therapy; therefore, WBRT has been widely used as a palliative treatment for metastatic brain tumors [93]. However, in recent years, remarkable advances have been made in systemic treatment, particularly in the field of lung cancer, and the control rate of extracranial lesions has improved. Consequently, the importance of controlling brain metastases has increased, and stereotactic radiosurgery (SRS) is often used to control intracranial disease. Brain metastases are considered relatively resistant to drug therapies because of the presence of the blood–brain barrier, making radiotherapy a critical treatment modality [94]. Previous randomized phase II studies for oligometastasis excluded patients with only intra-cranial oligometastasis. For example, Gomez et al. included only patients with brain metastases that had undergone prior local treatment [3]. SABR-COMET trial excluded the patients with only brain metastases [6]. Therefore, the number of oligometastasis to the brain is not clearly defined. The American Society for Radiation Oncology (ASTRO)/ESTRO clinical practice guideline for the treatment of oligometastasis [95] indicated that the management of brain metastases are considered under the 2022 ASTRO guidelines on brain metastases because of the additional complexity involved in decision-making [96]. This article provides an overview of single- and multifraction SRS for the treatment of brain metastases from lung cancer.

The ASTRO Clinical Practice Guidelines on brain metastases [96] present a structured treatment algorithm. SRS is strongly recommended for patients with solitary brain metastases and well-controlled extracranial disease. In cases involving metastases > 40 mm or those associated with neurological symptoms due to edema or hemorrhage, treatment options include surgical resection, WBRT, SRS, or a combination of WBRT and SRS. Post-operative radiotherapy is recommended to reduce the risk of local recurrence. Although evidence regarding the optimal radiation field and dose fractionation postoperatively is limited, a randomized trial comparing postoperative WBRT with SRS found no significant difference in OS [97]. WBRT showed better intracranial control, but was associated with a higher risk of cognitive dysfunction, leading to an increased preference for SRS. The ASTRO guidelines now strongly recommend postoperative SRS for local control [96].

Even when surgery is not possible, SRS is increasingly used in patients with up to four brain metastases and an ECOG performance status of 0–2. Randomized controlled trials show that the 1-year local control rate for brain metastases (≤4 lesions, ≤30 mm) treated with SRS is approximately 70–90% (Table 4) [98,99,100,101,102,103,104,105,106,107]. Emerging evidence suggests that SRS may be effective for up to 10 lesions. A prospective study by Yamamoto et al. involving 1194 patients (76% with lung cancer, total tumor volume ≤ 15 mL) reported a median survival time of 10.8 months in both the 2–4 and 5–10 metastatic groups without significant difference [108]. Furthermore, a post hoc analysis showed that cognitive decline and grade ≥ 3 adverse events were not significantly different between the two groups [109].

Recommended SRS dose regimens, based on tumor size, are provided in the ASTRO guidelines [96]: the recommended single-fraction SRS dose according to tumor size is as follows: ≤20 mm: 20–24 Gy, 21–30 mm: 18 Gy, 31–40 mm: 15 Gy. If the volume of normal brain receiving ≥12 Gy (V12 Gy) exceeds 10 cm^3^, multifraction SRS is recommended, as several studies have shown a significantly increased risk of brain necrosis above this threshold [110,111,112,113]. Tumors > 40 mm are generally better managed with surgery, but there is insufficient evidence to support the use of SRS for larger tumors [96].

An American Association of Physicists in Medicine Working Group task force review reported that the 1-year local control rate for tumors < 20 mm treated with 24 Gy was 95%, for tumors 21–30 mm treated with 18 Gy was 75%, and for 31–40 mm tumors treated with 15 Gy was 69% [114]. When treated with multifraction SRS (27–35 Gy in 3–5 fractions), local control was approximately 80%. A comparative study found that for tumors > 20 mm, multifraction SRS (27 Gy in three fractions) had better local control than SRS (15–18 Gy in a single-fraction), with 1-year control rates of 91% and 77%, respectively (*p* = 0.01) [115]. For optimal SRS outcomes, a BED_10_ of ≥50 Gy is recommended [116]. Inoue et al. found that V14 Gy (single-fraction) is equivalent to V23.1 Gy (three fractions) or V28.8 Gy (five fractions), with low necrosis risk when volumes are <7 cm^3^ [117]. Corticosteroids (dexamethasone, 4–8 mg daily) are typically the first-line treatment for patients with symptoms associated with radiation-induced brain necrosis [118]. Furthermore, bevacizumab may be particularly effective in patients who are resistant to steroids, have deep-seated disease, and have multiple necrotic brain lesions [119].

The combination of brain radiotherapy with molecularly targeted agents or ICI has raised safety concerns. Randomized data on the combinations of these targeted therapies or ICI with SRS are limited, and clinical decisions should be made with caution and careful consideration. According to recent reports, the combination of trastuzumab deruxtecan, a human epidermal growth factor receptor 2 (HER2)-targeted antibody-drug conjugate, and brain radiotherapy (WBRT or SRS) for brain metastases results in a significantly increased incidence of symptomatic brain necrosis (27.4% vs. 7.0%, *p* = 0.014) [120]. Therefore, the combination of antibody-drug conjugate agents and SRS should be avoided. Several retrospective studies have reported favorable local control rates and low necrosis rates with the combination of TKI and SRS [121,122,123]. However, concurrent TKIs and SRS have been reported to increase the cumulative incidence of brain necrosis over 12 months, and this trend is particularly pronounced when combined with anti-vascular endothelial growth factor receptor-TKIs, EGFR-TKIs, and HER2 antibodies [124]. A meta-analysis showed that the combination of SRS and ICI improved survival but was associated with higher complication rates [125]. Two retrospective studies have reported higher symptomatic radiation necrosis rates of 12–20% in patients treated with SRS and ICI [126,127]. Currently, a phase I study, DARREMI, is ongoing to investigate the safety of dose-reduced SRS in the treatment of brain metastases with concomitant ICI [128]. This study is expected to determine the optimized radiation dose for the patients receiving ICI.

In summary, brain metastases are considered resistant to chemotherapy because of the presence of the blood–brain barrier, making radiotherapy a critical treatment modality to control brain metastasis. Adequate management of brain metastases requires careful consideration of individual patient factors, including the number, size, and location of lesions, patient performance status, age, tumor histology, status of extracranial disease, available systemic therapy options, and treatment goals. Furthermore, it is necessary to investigate safe and effective methods of combining SRS with systemic therapy.

## 9. Conclusions and Future Directions

In this review, we summarize the local therapy, particularly SBRT, for oligometastatic lung cancer in several metastatic sites. We hope that this manuscript will be helpful for the use of SBRT in the treatment of oligometastatic lung cancer. The safety of additional SBRT also requires careful consideration because a clinical trial has reported that SBRT resulted in 5% treatment-related deaths [6]. Adverse events of SBRT that may impair patients’ daily activities or threaten the continuation of chemotherapy should be avoided, as systemic therapy remains the primary treatment for oligometastatic disease. ESTRO and EORTC OligoCare consortium reported an international Delphi consensus process among 28 interdisciplinary experts [22]. This consensus showed a potential need for and length of interruption to targeting therapy or ICI around SBRT delivery. Results of this systematic review and consensus process compile the best available evidence for the safe combination of metastases-directed SBRT and targeted therapy or ICI. Therefore, clinicians should consider this consensus to perform combined therapy with SBRT and these drugs safely.

Basic research studies have shown that SBRT releases tumor antigens into the blood and activates the immune system [129]. Therefore, SBRT is considered to promote the antigen–antibody response and enhance the efficacy of immunotherapy, and the combination of SBRT and ICI has been shown to improve prognosis in several stages of NSCLC, including oligometastatic disease [130,131,132,133,134]. Single-arm phase II trial was performed for 51 oligometastatic NSCLC patients with four or fewer metastases treated with local ablative therapy and pembrolizumab given sequentially [133]. The authors concluded that pembrolizumab after SBRT for oligometastatic NSCLC appears to improve PFS without compromising quality of life. While, when SBRT and ICI are performed, it is unclear whether the same dose as curative irradiation is necessary, the appropriate timing for combined use, and the number of tumors that should be irradiated. Currently, several clinical trials using SBRT combined with ICI are ongoing to enhance the immune activation effects for NSCLC including oligometastatic disease [135,136,137]. Proton beam therapy, which can reduce the wide-spread low dose area, is also an attractive combination therapy with ICI, because previous studies have shown that immune activity is affected by the irradiation dose to the blood pool [138].

Few studies have shown the efficacy of local consolidative therapy by specific sites of metastases. A retrospective observational study to compare pembrolizumab with and without local consolidative therapy for synchronous oligometastatic NSCLC indicated that survival benefit was reserved for local therapy to brain and lung metastases, without apparent benefit to bone metastases [135]. Further studies are warranted to identify patient populations that may benefit from local consolidative therapy at several oligometastatic sites. Medical oncologists, surgeons, and radiation oncologists should discuss the feasibility of local treatment and the continuation of systemic therapy for oligometastatic diseases at each facility.

A limitation of this manuscript was that a narrative review was performed, but not a systematic review. Unlike systematic reviews, narrative reviews do not follow strict protocols for literature searching and selection. While, narrative reviews can include a wide variety of studies and provide an overall summary, with interpretation and critique [139]. Furthermore, in this manuscript, we have discussed dose fractionation of SBRT in several metastatic sites; however, individualized treatment planning is also important. An optimal treatment plan should be performed based on the patient and tumor characteristics, such as previous treatment history, comorbidities, tumor size, location, and the biological behavior.

Currently, “JCOG2108: a multicenter randomized phase III study of systemic chemotherapy followed by maintenance therapy versus local consolidation therapy for postoperative oligometastatic recurrent non-small cell lung cancer” is ongoing in Japan. A randomized controlled trial is currently underway to verify the significance of adding radical local therapy, including surgery and SBRT, to the treatment of oligometastatic lung cancer. High-quality prospective clinical trials on oligometastases are expected in Japan. Further studies are warranted to clarify aspects such as the applicable population for combined treatment, treatment timing, dose optimization, efficacy evaluation indicators, and exploration of potential biomarkers for safe and effective radiotherapy for oligometastatic NSCLC.

## Figures and Tables

**Table 1:** Previous retrospective studies on the comparison between SBRT and other treatment modalities for pulmonary metastases.

Author	Year	Patient Number	Lung Cancer (%)	Treatment Modalities	Overall Survival	Local Control	Grade ≥ 3 AEs
Lee [14]	2018	21	13.3%	SBRT (60 Gy in 3 fr or 48 Gy in 4 fr)	2 y: 68.2%	2 y: 75.3%	4.8%
			9.5%	Wedge resection, lobectomy	2 y: 81.8%	2 y: 91.5%	10.0%
Kanzaki [15]	2020	82	N/A	SBRT (52 Gy in 4 fr or 60 Gy in 10 fr)	3 y: 52%	3 y: 92%	N/A
			N/A	Wedge resection, segmentectomy, lobectomy	3 y: 77%	3 y: 88%	N/A
Gits [11]	2024	644	N/A	SBRT	2 y: 63.3% *	N/A	2.6%
			N/A	Sublobar resection	2 y: 80.3% *	N/A	2.0%
			N/A	Percutaneous thermal ablation	2 y: 83.8% *	N/A	2.4%
Song [16]	2024	54	0% (Sarcoma)	SBRT (48–60 Gy in 3–10 fr)	3 y: 57.3% *	3 y: 92.3%	0%
				Wedge resection, segmentectomy, lobectomy	3 y: 84.6% *	3 y: 92.9%	N/A
Wang [12]	2025	335	0% (CRC)	SBRT (30–70 Gy in 1–10 fr)	3 y: 78.9% *	N/A	0%
				Wedge resection, segmentectomy, lobectomy	3 y: 85.9% *	N/A	0%
Shin [17]	2025	209	0% (HCC)	SBRT (40–60 Gy in 3–6 fr)	2 y: 83.0% *	2 y: 97.8%	0%
				Wedge resection, segmentectomy, lobectomy	2 y: 72.6% *	2 y: 98.0%	0%

AE, adverse event; fr, fractions; y, year; N/A, not applicable; CRC, colorectal cancer; HCC, hepatocellular carcinoma; SBRT, stereotactic body radiotherapy; Gy, Gray. * Comparison using propensity score-based adjustments.

**Table 2:** Previous reports on SBRT for liver metastases.

Author	Year	Trial	Patient (Tumor) Number	Lung Cancer (%)	Dose Prescription	Follow-Up	Overall Survival	Local Control	Grade ≥ 3 AEs
Rusthoven [34]	2009	Phase 1/2	47 (63)	10	36–60 Gy in 3 fr	16 m	Median: 20.5 m	1 y/2 y: 95%/92%	<2% with no RILD
Lee [40]	2009	Phase 1	68 (143)	2	27.7–60 Gy in 6 fr	11 m	Median: 17.6 m	1 y: 71%	10% with no RILD
Scorsetti [33]	2018	Phase 2	61 (76)	N/A	52.5–75 Gy in 3 fr	61 m	1 y/3 y: 85.2%/31.1%	1 y/3 y: 94%/78%	1.6%
Folkert [41]	2021	Phase 1	33 (39)	N/A	35–40 Gy in 1 fr	26 m	2 y: 82%	4 y: 96.6%	0%
Mahadevan [42]	2018	Retrospective	427 (568)	52	45 (12–60) Gy in 3 (1–5) fr	14 m	Median: 22 m	Median: 55 m	N/A

AE, adverse event; fr, fractions; m, months; y, year; N/A, not applicable; RILD, radiation-induced liver disease; SBRT, stereotactic body radiotherapy; Gy, Gray.

**Table 3:** Previous reports on SBRT for adrenal oligometastases from lung cancer.

Author	Year	Patient Number	Dose Prescription	Overall Survival	Local Control	Grade ≥ 3 AEs
Holy [60]	2011	13	15–40 Gy in 3–6 fr	Median: 23 m	Overall: 77%	N/A
Gamsiz [61]	2015	15	30 Gy in 3 fr	16 m: 33%	16 m: 87%	0%
Zhao [62]	2018	32	32–50 Gy in 3–8 fr	1 y: 58%	1 y: 97%	0.3%
Arcidiacono [63]	2020	37	30–50 Gy in 5 fr	2 y: 68%	2 y: 54%	0%
Rzazade [64]	2022	44	45–50 Gy in 5 fr	2 y: 57%	2 y: 91%	0%

AE, adverse event; N/A, not applicable; fr, fractions; m, months; y, year; Gy, Gray.

**Table 4:** Results of RCT of stereotactic radiotherapy for brain metastases.

Author	Year	Patient Number	Lung Cancer (%)	BMs	Dose Prescription	Overall Survival	Local Control	Intracranial Progression-Free Survival	Radiation Necrosis
Aoyama [98]	2006	67	67%	1–4 (<3 cm)	<2 cm: 22–25 Gy in 1 f >2 cm: 18–20 Gy in 1 fr	1 y: 28.4%	91%	1 y: 23.6%	Grade 4: 1.5%
Muacevic [99]	2008	31	32.3%	1 (<3 cm)	14–27 Gy in 1 fr	Median: 10.3 m	1 y: 96.8%	1 y: 74.2%	Grade 4: 3.2%
Chang [100]	2009	30	43%	1–3	15–20 Gy in 1 fr	Median: 15.2 m 1 y: 63%	1 y: 67%	1 y: 27%	Grade 4: 6.7%
Aoyama [101] Aoyama [102]	2007 2015	45	100%, excluding SCLC	1–4 (<3 cm)	<2 cm: 22–25 Gy in 1 fr >2 cm: 18–20 Gy in 1 fr	Median: 8.6 m 1y: 28.4%	91%	Median: 6.2 m 1 y: 23.6%	Grade >3: 2.2%
Lim [103]	2015	49	100%, excluding SCLC	1–4 (<3 cm)	NA	Median: 14.6 m 1 y: 57%	1 y: 84.6%	Median: 9.4 m 1 y: 44.1%	N/A
Brown [104] Churilla [105]	2016 2017	111	72.1%	1–3 (<3 cm)	<2 cm: 24 Gy in 1 f >2 cm: 20 Gy in 1 fr	Median: 10.4 m 1 y: 31.7%	1 y: 72.8%	1 y: 50.5%	Grade >3: 1.8%
Bodensohn [106]	2023	40	60%, excluding SCLC	4–10 (<2.5 cm)	15–20 Gy in 1 fr	Median: 10.4 m 1 y: 48.2%	100%	Median: 7.1 m	Grade >3: 0%
Scorsetti [107]	2023	251	57%, excluding SCLC	1–4 (<3 cm)	≤2 cm: 24 Gy in 1 f >2.1–3 cm: 20 Gy in 1 fr	1 y: 65.7%	1 y: 98.2%	1 y: 63.5%	Grade 3: 4.8%

BMs, brain metastases; y, year; SCLC, small-cell lung cancer; fr, fraction; N/A, not applicable; RCT, randomized controlled trial; m, months; y, year; Gy, Gray.

## Abbreviations

The following abbreviations are used in this manuscript:

EORTC	European Organization for Research and Treatment of Cancer
SBRT	Stereotactic body radiotherapy
NSCLC	Non-small cell lung cancer
PFS	Progression-free survival
HR	Hazard ratio
CI	Confidence interval
OS	Overall survival
TKI	Tyrosine kinase inhibitor
EGFR	Epidermal growth factor receptor
BED	Biologically effective dose
PTV	Planning target volume
SCLC	Small cell lung cancer
ECOG	Eastern Cooperative Oncology Group
CT	Computed tomography
MRI	Magnetic resonance imaging
FDG-PET	18-fluoro-deoxyglucose positron emission tomography
ESTRO	European Society for Radiotherapy and Oncology
ICI	Immune checkpoint inhibitor
CCI	Coverage Compromise Index
WBRT	Whole-brain radiation therapy
SRS	Stereotactic radiosurgery
ASTRO	American Society for Radiation Oncology
HER2	Human epidermal growth factor receptor 2
